# The impact of a brief RNR‐based training on Turkish juvenile probation officers' punitive and rehabilitative attitudes and recidivism risk perceptions

**DOI:** 10.1002/jcop.22310

**Published:** 2019-12-26

**Authors:** Ayşe E. Tuncer, Gizem Erdem, Corine de Ruiter

**Affiliations:** ^1^ Department of Clinical Psychological Science Maastricht University Maastricht The Netherlands; ^2^ Koç University Istanbul Turkey

**Keywords:** juvenile probation officers, punitive attitudes, recidivism risk perception, rehabilitative attitudes, risk‐need‐responsivity model

## Abstract

The present quasi‐experimental study examined the impact of a brief training program based on the risk–need–responsivity (RNR) model on Turkish juvenile probation officers' (JPOs) punitive and rehabilitative attitudes toward justice‐involved youth and recidivism risk perceptions. Fifty‐nine JPOs were recruited through three probation offices in Istanbul, Turkey. Thirty‐six JPOs, who received a 1‐day training in the RNR model of offending behavior, were compared to JPOs in a wait‐list control condition (*n* = 23). Participants in both conditions completed surveys at baseline and 1‐week posttraining. Mixed‐factorial analysis of variances revealed a significantly higher decrease in JPOs' punitive attitudes from pre‐ to posttest, in the training condition compared to the control group, with a medium effect size. Rehabilitative attitudes decreased in both conditions, while recidivism risk perceptions did not change from pre‐ to posttest in either condition. Future research could expand on these promising results using a more intensive training program and a randomized‐controlled design in a larger sample of JPOs.

## 1 INTRODUCTION

The probation system in Turkey was established in 2005 (Yavuz, 2012) and the number of youth sentenced to probation has shown a sharp increase since then. According to the Turkish Ministry of Justice (TMJ, 2018), there are currently around 200,000 cases under probationary supervision, 13,646 of which are youth between ages 12–18 years. The majority of justice‐involved youth in Turkey is convicted of drug offenses (primarily drug possession) and shows significant needs for treatment and rehabilitation (Kocagazioğlu, Işıker, & Demircan, 2016).

Currently, around 4,000 probation officers (POs) supervise the increasing number of probationers in Turkey (Işık, 2016). Youth under probation are primarily assigned to 1,600 POs who have attended a limited number of brief professional training programs on substance abuse, anger management, and motivational interviewing. These POs are referred to as juvenile probation officers (JPOs), although none of their training programs are specifically designed to address the unique needs of youth under probation, nor are they based on research evidence regarding effective methods in reducing recidivism (Işık, 2016). Next to a lack of specific evidence‐based training, officers face the inherent challenges of being a PO, including conflict between their rehabilitation and law enforcement role (Miller, 2015). Meanwhile, JPOs are the backbones of the probation system that strives to offer community‐based services to decrease youth's risk of recidivism. The JPOs oversee the correctional plan of the youth as ruled by the juvenile court. The standard probation period for youth lasts 1 year, during which youth are mandated to attend individual sessions, group sessions, and seminars. Depending on the individualized correctional plan of the youth, the frequency and duration of these sessions vary from 3 to 12 meetings over a year, with additional home visits or community service. The ultimate goal of the juvenile probation system is to execute a youth's sentence in the community and to lower the risk of future offending (TMJ, 2018).

Given that the probation system is relatively new in Turkey, there is a lack of research on youth recidivism rates as well as antecedents to youth justice involvement. In other countries, including the US and European countries, a wide array of research has been conducted to identify the risk factors for reoffending. Moffitt (1993) has proposed a distinction that has implications for understanding these factors and developing effective interventions. According to Moffitt (1993), the first category is individuals who show antisocial behavior only during adolescence and do not repeat the behavior. The second category of individuals shows a lifetime‐persistent‐offending pattern as a result of the interaction between exposure to criminogenic environments as a child and childhood neuropsychological problems. (Moffitt, 1993). Longitudinal research has also shown that individuals with a relatively early onset of aggression, delinquency, and substance use and are more likely to continue offending into adulthood (de Ruiter & Augimeri, 2012). The distinction between adolescence‐limited and lifetime‐persistent‐offending patterns is a valuable tool for JPOs to decide the type, duration, and intensity of intervention programs to prevent youth reoffending.

Several studies focused on the implementation of intervention programs for youth in probation and have widely documented the ineffectiveness of punishment‐oriented approaches in reducing youth recidivism (Lipsey, 2009; MacKenzie & Farrington, 2015; Nagin, Cullen, & Jonson, 2009; Petrosino, Turpin‐Petrosino, & Guckenburg, 2010; Rhine, Mawhorr, & Parks, 2006). In a meta‐analysis of 548 intervention studies, Lipsey (2009) found an 8% increase in recidivism rates when punitive approaches were employed. On the other hand, rehabilitation‐oriented interventions such as counseling and skill‐building have resulted in a 10–13% reduction in recidivism. MacKenzie and Farrington (2015) examined the findings of randomized‐controlled trials, systematic reviews, and meta‐analyses performed between 2005 and 2015 on justice‐involved youth, and found that interventions based on punitive approaches, such as deterrence and discipline, are ineffective, while rehabilitative approaches are effective in preventing future offending (Koehler, Lösel, Akoensi, & Humphreys, 2012). Among these rehabilitative programs, the risk–need–responsivity (RNR) framework, that uses a collaborative supervision model focused on individualized treatment plans for the youth involved in crime, has the most empirical support (Andrews & Bonta, 2010; Vincent, Guy, Perrault, & Gershenson, 2016; Vitopoulos, Peterson‐Badali, & Skilling, 2012). In a meta‐analysis study to examine the effects of young rehabilitation programs on 7,940 justice‐involved youth, Koehler et al. (2012) found the most substantial mean effect in RNR groups (odds ratio = 1.90) indicating a 16% decrease in recidivism against a baseline of 50%.

The Turkish probation system currently lacks an evidence‐based intervention and training framework, and the current study is a first step to address this need. In an attempt to introduce a rehabilitation‐oriented probationary supervision approach for Turkish youth under probation, we designed a 1‐day RNR‐based training to initiate an attitudinal change in JPOs. It was especially important to target the JPOs and engage them in a training program because they serve as active agents of change and their values and attitudes contribute to the goal of the probation system (Werth, 2013). It has been consistently found that officer attitudes make a difference in recidivism outcomes (Kennealy, Skeem, Manchak, & Eno Louden, 2012) and their importance has been emphasized by the RNR framework (Dowden & Andrews, 2004). Therefore, the content of the 1‐day program was built on the premise that the first step toward changing juvenile delinquent behavior lies in changing JPO's attitudes (Vincent, Paiva‐Salisbury, Cook, Guy, & Perrault, 2012). The content focused on emphasizing the effectiveness of rehabilitative approaches in decreasing youth recidivism, adolescence‐limited versus lifetime persistent offending, review of RNR principles, intervention techniques targeting criminogenic risk factors, such as anger management and substance abuse, and basic communication skills for the JPOs (see below for more detailed information on the content of the training program). Consistent with Vincent et al.'s (2012) study, we expected that informing the JPOs about the trajectories of juvenile offending would help JPOs understand that youth recidivism risk is low. Therefore, we expected their recidivism risk perceptions would decrease.

The current study examined the effect of brief RNR training on JPO's attitudes and recidivism risk perceptions. We hypothesized that the difference between pretest and posttest scores on attitudes and recidivism perceptions would be significantly higher in the RNR training group than the control group. That is, JPOs in the intervention condition would show significantly less punitive attitudes, more rehabilitative attitudes, and lower recidivism risk perceptions from pre‐ to posttest than those in the control condition.

## 2 Method

### 2.1 Study design

This study was designed as a pilot study (Stage I nonrandomized trial; Rounsaville, Carroll, & Onken, 2001) to develop and test the immediate impact of the RNR‐based training program for JPOs in Istanbul, Turkey. The stage model of behavioral interventions provides guidelines for intervention development and program evaluation in human subjects with specific design considerations for each phase of the intervention's development. Consistent with the recommendations of Rounsaville et al. (2001), as well as the standards of evidence of the Society for Prevention Research (Gottfredson et al., 2015), our Stage I study focused on the needs assessment of participants via qualitative focus groups, development of a standardized manual, and training material, followed by a quantitative quasi‐experimental study of the initial impact of the program using a small sample. A Stage I design was preferred over a Stage II design because there were no Turkish training materials available for an RNR‐based intervention program nor any empirical studies examining the effects of RNR‐based training in the Turkish criminal justice system. The needs assessment and program development phases of the study are available elsewhere (Erdem et al., 2019) while the current paper focuses on the quantitative aspects of the quasi‐experimental study.

### 2.2 Recruitment and participants

Sampling and recruitment procedures started after obtaining official permission from the Turkish Ministry of Justice as well as from the directors of three probation directorates in Istanbul. Data were collected in May 2017 through three probation offices in Istanbul. Study sites were located in the urban areas of Istanbul, covering three regions of the city (i.e., Asian side, European side, and the historical peninsula), serving both adults and youth under probation. They were comparable in terms of staff numbers and size (33, 29, and 34 JPOs per probation office, respectively).

Participation in the research project and the training was voluntary. To be eligible, participants had to be currently working with at least one youth under probation and be committed to complete both assessments at pretest and posttest.1Participants who confirmed their availability to complete both assessments and the training were recruited for the study because there was significant mobility of POs across sites and cities at the time of data collection. Eligible JPOs willing to participate in the study were recruited upon providing written informed consent. To ensure voluntary participation, supervisors were not involved in the recruitment process. Besides, the participants were informed about their right to skip survey questions and to withdraw from the study at any time.

The study employed a quasi‐experimental pretest posttest intervention design with a wait‐list (no‐training) control group. All JPOs across three sites (*n* = 96) were invited to the study and 59 officers agreed to participate (participation rate: 64.1%). The most common reasons for not participating were annual or maternity leave, having to attend another training, and a high workload. The total sample included 59 JPOs with 22 participants from Office 1 (training condition), 14 participants from Office 2 (training condition), and 23 participants from Office 3 (control condition).

The demographic characteristics of the sample are available in Table 1. Of the 59 participants, the majority were women (*n* = 49, 83.1%) and had a college degree (*n* = 40, 67.8%). The average age was 31.31 years (standard deviation [*SD*] = 4.37; range = 26–44). Participants reported 5.9 years (*SD* = 2.93; range = 1–15) of work experience in the criminal justice system on average. The two conditions did not differ in terms of gender, age, and years of work experience in the probation system. All participants in the training condition attended all RNR modules (100% attendance rate) and completed the surveys. All participants in the control condition completed the pretest while three of the 23 participants missed the posttest survey (13% attrition rate).

**Table 1:** Descriptive features of the training and control groups (percentages, mean, and standard deviations)

	Total *N* = 59		Training condition *n* = 36	Control condition *n* = 23	
Variable	*n* (%)	*M* (*SD*)	*n* (%)	*n* (%)	Test
Gender					
Male	10 (16.9%)		4 (11.1%)	6 (26.1%)	*χ* ^2^ (1) = 2.24, *p* = .13
Female	49 (83.1%)		32 (88.9%)	17 (73.9%)	
Education level					
Bachelor degree	40 (67.8%)		28 (77.8%)	12 (52.2%)	
Graduate degree	19 (32.2%)		8 (22.2%)	11 (47.8%)	*χ* ^2^ (2) = 4.14, *p* = .04
Age		31.31 (4.37)	31.42 (4.67)	31.13 (3.96)	*t* (57) = −0.24, *p* = .81
Years of experience in criminal justice		5.90 (2.93)	5.55 (2.54)	6.43(3.43)	*t* (57) = 1.13, *p* = .26

Abbreviation: *SD*, standard deviation.

### 2.3 Procedure

Upon obtainment of written informed consent, participants continued with the pretest assessment. The survey included a demographic questionnaire, scales that assessed attitudes toward justice‐involved youth, and JPOs' perceptions of the likelihood of recidivism. Participants in both conditions spent approximately 10 min completing the survey. After the initial assessment, the JPOs in the training condition received the 1‐day RNR‐based training while those in the control condition were wait listed. Both groups completed the posttest survey 1 week after the pretest. Participants in the control condition received the RNR‐based training after the study was completed. The Turkish Ministry approved the present study with protocol number 46985942/151/18410. Ethical approval was obtained from the Institutional Review Board (IRB) of Koç University with protocol number 2017.009.IRB3.005.

### 2.4 The RNR‐based training

The principles of the training conformed with Bonta's et al. (2011) and Vincent et al.'s (2012) recommendations. The training exercises, cases, and other materials were tailored to match the needs of Turkish POs, based on the findings of a prior qualitative study conducted by the first and second authors (Erdem et al., 2019). A series of focus groups with a separate sample of POs and administrative staff in Istanbul revealed primary needs for training in interviewing skills and risk assessment as well as preference for brief interventions. While the training adhered to the RNR model and principles in the current study, the curriculum was limited to 1 day, as opposed to the 3‐day training program of the original RNR approach. Our training consisted of four 90‐min modules and it was conducted in the Istanbul Office of Probation by the first author (a clinical psychologist with more than 15 years of clinical and supervisory experience).

The first module comprised a review of the rationale for the training and an introduction to empirical research on rehabilitative versus punitive approaches to probation. The focus of the first module was to challenge common misconceptions (i.e., “once an offender, always an offender”) about justice‐involved youth by presenting findings from current research on youth delinquent behavior. In addition, the JPOs were informed about findings on the criminal trajectories and reoffending risk of justice‐involved youth (e.g., Constantine, Andel, Robst, & Givens, 2013; Sampson & Laub, 2003). The module emphasized that delinquent behavior might be a passing phase in adolescence (Moffitt & Caspi, 2001). Youth recidivism statistics from the US and Europe were shared, because there were no data yet regarding youth recidivism rates in Turkey. Providing youth‐specific information was necessary because most Turkish JPOs are working with adults as well as youth in probation. We wanted to make clear that the needs of youth under probation are not just different in quantity from adults, but also in quality.

The second module consisted of a review of the RNR principles and group work on brief case descriptions that the JPOs had been asked to bring to the training. Participants practiced intervention skills to address the needs of justice‐involved youth, such as anger management, substance abuse, and self‐regulation. The third module focused on the responsivity factor and the working alliance and collaboration with justice‐involved youth. Participants were engaged in role‐play activities to practice active listening, reflection, and empathic confrontation skills, as well as other communication skills to overcome resistance among the youth. The cognitive behavioral therapy model of the link between thoughts and emotions (Beck & Beck, 2011) was the focus of the fourth module. According to this model, our thoughts have an impact on how we feel and behave. Therefore, by changing the way we think, we can manage our emotions and behavior. To this end, we practiced clinical skills such as generating alternative thoughts, guided discovery, and socratic questioning with the JPOs.

### 2.5 Measures

#### 2.5.1 Outcome variables

The dependent variables of this study were punitive attitudes, rehabilitative attitudes, and recidivism risk perceptions, assessed by the correctional goal scale, the support for rehabilitation scale, and recidivism risk perception score, respectively. The first author translated all measures from English into Turkish and a bilingual translator subsequently back‐translated to English. The two translators finalized the Turkish translation after reaching consensus.

#### 2.5.2 The correctional goal scale

This is a 7‐point Likert‐type scale with six items, developed to measure attitudes toward the criminal sanctioning of adults (Cullen, Clark, Cullen, & Mathers, 1985). The internal consistency was reported 0.63 in a prior study (Cullen et al., 1985). The version used in the present study employed a 5‐point Likert‐type scale ranging from 1 (*strongly disagree*) to 5 (*strongly agree*) to measure punitiveness toward justice‐involved youth, derived from Vincent et al. (2012). The internal consistency in our sample was 0.70 at the pretest.

#### 2.5.3 The support for rehabilitation scale

The scale comprises six items rated on a 7‐point Likert‐type scale to assess support for rehabilitation of justice‐involved youth (Bazemore & Feder, 1997). It yielded a Cronbach's *α =* .79 in the original study (Bazemore & Feder, 1997). For the present study, the adapted version with a 5‐point Likert‐type scale ranging from 1 (*strongly disagree*) to 5 (*strongly agree*) was used (Vincent et al., 2012). The reliability coefficient was 0.62 for our sample at the pretest.

#### 2.5.4 Recidivism risk perception score

Recidivism risk perception was assessed with one question from Vincent et al.'s (2012) study: “What percentage of the youth you work with do you think are likely to re‐offend?” Participants were asked to give a number ranging from 0 to 100.

We used the scales adapted by Vincent et al. (2012) because this study was similar in terms of the measured‐dependent variables (punitive attitudes, rehabilitative attitudes, and recidivism risk perceptions). Additionally, there were no standardized measures for Turkish youth probationers and we decided to employ validated measures from a similar study for comparative purposes. There are no external validity studies for the original scales.

### 2.6 Data analysis

Initial analyses focused on the comparison of demographic characteristics and outcomes of training and control groups at baseline, to examine the compatibility of the two groups. Independent samples *t* tests were run to compare continuous variables (years of experience in the criminal justice system, punitive and rehabilitative attitudes, and perception of recidivism), and *χ* ^2^ tests were conducted for categorical variables (gender, education level) to examine group differences at baseline. For the main analysis, this study goal was to examine the amount of change in punitive attitudes, rehabilitative attitudes, and recidivism risk perception from pre‐ to posttest as a function of intervention condition. Therefore, we ran a mixed‐factorial analysis of variance (ANOVAs) for each dependent variable with time (pretest vs. posttest) as a within‐subjects factor and group (intervention vs. control) as a between‐subjects factor. The analysis examined the main effects of time (pretest and posttest), main effects of condition (intervention vs. control), and interaction effects (time × condition). The interaction effects on the dependent variables were the main analyses of interest, because we expected a higher impact of the intervention condition, as compared to the control condition. Cohen's *d* was calculated as a measure of effect size.

## 3 RESULTS

### 3.1 Comparison of groups at baseline

The demographic characteristics of the training and control groups were compared at pretest (see Table 1). There were no significant differences between groups in participants' gender [ *χ* ^2^ (1) = 2.24, *p* = .13], age [*t* (57) = −0.24, *p* = .81], years of work experience [*t* (57) = 1.13, *p* = .26], or size of caseload [*t* (48) = 0.86, *p* = .39]. An exception was education level; a higher proportion of participants in the control condition had a graduate degree, compared to those in the training group [ *χ* ^2^ (1) = 4.14, *p* = .04; Table 1]. We subsequently conducted independent *t* tests to examine whether officers with a college versus graduate degree differed on the dependent variables of interest. There were no differences between groups in punitive attitudes [*t* (57) = 0.15, *p* = .88], rehabilitative attitudes [*t* (57) = −0.96, *p* = .34], or recidivism perception at baseline [*t* (57) = 0.76, *p* = .45]. Therefore, educational level was not included in subsequent analyses as a control variable. Independent samples *t* tests revealed no significant differences between training and control groups in punitive attitudes [*t* (57) = 1.31, *p* = .20], rehabilitative attitudes [*t* (57) = 0.40, *p* = .69], and recidivism risk perceptions [*t* (57) = 0.15, *p* = .89] at pretest.

### 3.2 Main analyses

#### 3.2.1 Punitive attitudes

We present mixed‐factorial ANOVA results in Table 2. The interaction of group and pre‐post test scores was our main target of interest, but we first tested for main effects. We did not find a main effect for the time [*F* (1, 54) = 2.35, *p* = .13] nor for the condition [*F* (1, 54) = 0.59, *p* = .44] on punitive attitudes. We found a significant interaction effect on punitiveness [*F* (1, 54) = 4.48, *p* = .04, Cohen's *d* = −0.60], revealing that JPOs in the training condition reported a significantly larger decrease in punitive attitudes than JPOs in the control condition.

**Table 2:** Mean and standard deviations on punitive attitudes, rehabilitative attitudes, and recidivism risk perception for training and control groups at pre‐ and posttest and the mixed‐factorial ANOVA interaction effects

	Training condition		Control condition		Interaction effect		Cohen's
	Pretest	Posttest	Pretest	Posttest	(Time × group)	*P*	*d*
Punitive attitudes	2.19 (0.42)	1.99 (0.49)	1.98 (0.73)	2.0 (0.70)	*F* (1, 54) = 4.48	.04	0.60
Rehabilitative attitudes	3.87 (0.41)	3.81 (0.47)	3.92 (0.55)	3.80 (0.46)	*F* (1, 54) = 0.80	.37	0.25
Recidivism risk perception	53.30 (12.57)	51.15 (10.64)	52.52 (23.71)	53.0 (21.73)	*F* (1, 54) = 0.20	.65	0.13

Abbreviation: ANOVA, analysis of variance.

#### 3.2.2 Rehabilitative attitudes

Regarding rehabilitative attitudes, we found a significant main effect of time [*F* (1, 54) = 4.41, *p* = .04], but not condition [*F* (1, 54) = 0.08, *p* = .77], indicating that rehabilitative attitudes significantly decreased in both groups, but the decrease was not significantly different between training and control conditions. There was no significant interaction effect for rehabilitative attitudes [*F* (1, 54) = 0.80, *p* = .37, Cohen's *d* = 0.25].

#### 3.2.3 Recidivism risk perception

**Figure 1:**
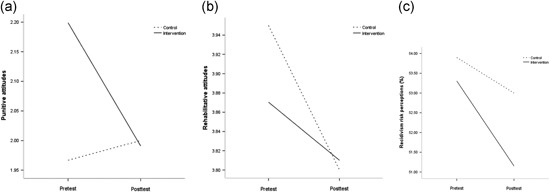
The interaction between RNR‐based training and (a) punitive attitudes, (b) rehabilitative attitudes, and (c) recidivism risk perceptions. RNR, risk–need–responsivity

There was no main effect of time [*F* (1, 54) = 1.21, *p* = .27], nor the main effect of the condition [*F* (1, 54) = 0.08, *p* = .78] or interaction effect [*F* (1, 54) = 0.20, *p* = .65, Cohen's *d* = 0.13] for recidivism risk perception. Figure 1 illustrates the means for punitive attitudes, rehabilitative attitudes, and recidivism risk perceptions by time (pretest and posttest) and condition (intervention vs. control).

## 4 DISCUSSION

The training was based on the RNR framework that offers a rehabilitative approach to supervision and was implemented in the primarily punitively oriented criminal justice system of Turkey with the second‐highest incarceration rate in Europe (World Prison Brief, 2018). We expected that exposure to an evidence‐based rehabilitative framework would impact JPOs' attitudes and recidivism risk perceptions. The JPOs in the RNR‐based training group reported significantly higher reductions in their punitive attitudes from pre‐ to posttest, compared to the control group. Contrary to our expectations, rehabilitative attitudes decreased from pre‐ to posttest in both groups, but groups did not differ by condition. There was also no interaction effect of training and time on recidivism risk perceptions.

A previous study with Turkish POs (some JPOs at the same time) found that officers struggled to implement a rehabilitative approach within the probation service due to barriers in coordination with other governmental agencies in referrals and limited availability of learning and rehabilitative programs for justice‐involved youth (Erdem et al., 2019). Therefore, officers stated they were primarily focused on short‐term practical solutions to youth's needs, rather than coordinating rehabilitative services. Our current findings align with and expand on those of Erdem et al. (2019), suggesting that a brief RNR‐based training has promising effects on reducing punitive attitudes, but not on promoting rehabilitative attitudes among JPOs. Unexpectedly, there was a decrease in JPOs' rehabilitative attitudes from pre‐ to posttest in both conditions. It appears that our RNR‐based training presented officers an opportunity to develop less punitive attitudes towards justice‐involved youth. However, the actual promotion of rehabilitative attitudes may have been less effective given the punitive orientation of the Turkish criminal justice system, coordination issues, and limited availability of rehabilitative programs. The finding may also be related to the low internal reliability of the support for rehabilitation scale.

Contrary to Vincent et al.'s (2012) findings, our RNR‐based training had no favorable impact on JPO's recidivism risk perceptions from pre‐ to posttest, as compared to the control condition. This finding could be due to differences in the research design of the two studies. Vincent et al.'s (2012) study was focused on the effects of the implementation of risk‐need assessment tools (specifically, the structured assessment of violence risk in youth and the youth level of service/case management inventory) in six youth probation offices and did not include control sites. The current study, on the other hand, focused on introducing the RNR framework as a rehabilitative framework in a justice system that currently lacks a standardized evidence‐based risk‐need assessment tool. The implementation of a structured risk assessment instrument might be a key factor for actual changes in rehabilitative attitudes and service provision to take place (Schwalbe, 2008).

### 4.1 Limitations of the present study

The findings of the current study should be interpreted with caution due to several limitations. The current study implemented a pre‐post test wait‐list control group design with a small sample size. The current study is underpowered, given that the minimum sample size to achieve 0.80 power at *α =* .05 is 100 participants, as suggested by Machin, Campbell, Tan, and Tan (2011). However, this is a Stage I study that examined the immediate effects of the RNR‐based training program on JPOs' attitudes and it is a common practice to test immediate effects in a small sample. Therefore, power is not the primary concern (Rounsaville et al., 2001). Given the promising effects of the pilot intervention on reducing punitive attitudes of JPOs, future research should employ a Stage II efficacy trial design, using a larger sample size (*N* > 100) and randomization, with longer term follow‐up postintervention.

Another limitation was the low internal reliability of the support for rehabilitation scale, indicating potential measurement error in assessment. Additionally, the absence of actual recidivism data of justice‐involved youth is a significant issue in the current study. We used outcome measures focused on change in JPOs' attitudes and recidivism risk perceptions only, rather than a change in behavioral outcomes of the justice‐involved youth. Additionally, the JPOs in the Turkish criminal justice system have high mobility (i.e., officers were reappointed to different units or institutions after training), limiting the opportunity to conduct long‐term follow‐up assessments. Thus, the current design does not examine the long‐term sustainability of program effects on punitive attitudes.

### 4.2 Implications for future research and practice

Overall, the findings of the current study suggest that the interplay between punitive versus rehabilitative attitudes towards justice‐involved youth is complex. It appears that punitive and rehabilitative approaches may not necessarily be mutually exclusive in the Turkish context because they may not be opposite ends of the same spectrum. That is, training that reduces punitive attitudes may not promote rehabilitative attitudes to the same extent. Prior research has also shown that POs may integrate rehabilitative and punitive attitudes in their supervision practices at varying levels (Miller, 2015). Still, RNR‐based training appears to have a promising effect in terms of reducing punitive attitudes among JPOs. Such training programs can provide JPOs with skills and resources to work with justice‐involved youth and promote less punitive approaches. Given the favorable outcomes of rehabilitative programs in reducing crime and recidivism rates among justice‐involved youth (MacKenzie & Farrington, 2015), future research should examine the ways in which training programs could enhance actual rehabilitative probation interventions, and ultimately recidivism rates in Turkey.

Furthermore, it was unexpected that rehabilitative attitudes decreased among the JPOs in both conditions. This finding may be pointing at a measurement problem. Thus, it calls for more research on how best to measure rehabilitative attitudes and endeavors for the development of more precise measures.

The current study has implications for youth probation practices in Turkey. The RNR‐based training was tailored to meet the needs of Turkish JPOs and materials are available for the JPOs to execute a more collaborative approach in their work with youth on probation. Our project also sparked the interest of UNICEF Turkey and the Turkish Ministry of Justice to expand and implement an RNR‐based approach in the youth probation system. In 2018, we launched a large‐scale country‐wide project to develop an RNR‐informed risk assessment protocol and a new rehabilitation system targeting 12‐ to 18‐year old youth on probation. The program is currently implemented in 24 probation offices across 21 cities in Turkey (TMJ, n.d.).

## 5 CONCLUSION

This study was the first to implement and evaluate RNR‐based training in the Turkish youth probation system. With limited means, we managed to demonstrate that Turkish JPOs' punitive attitudes could decrease via brief intervention. Future studies should explore the long‐term impact of RNR‐based training on JPO's attitudes, their real‐world interactions with justice‐involved youth, and the possible impact on recidivism reduction.
